# Differential Psychological Impact of Internet Exposure on Internet Addicts

**DOI:** 10.1371/journal.pone.0055162

**Published:** 2013-02-07

**Authors:** Michela Romano, Lisa A. Osborne, Roberto Truzoli, Phil Reed

**Affiliations:** 1 Università degli Studi di Milano, Milan, Italy; 2 Swansea University, Swansea, United Kingdom; MIT, United States of America

## Abstract

The study explored the immediate impact of internet exposure on the mood and psychological states of internet addicts and low internet-users. Participants were given a battery of psychological tests to explore levels of internet addiction, mood, anxiety, depression, schizotypy, and autism traits. They were then given exposure to the internet for 15 min, and re-tested for mood and current anxiety. Internet addiction was associated with long-standing depression, impulsive nonconformity, and autism traits. High internet-users also showed a pronounced decrease in mood following internet use compared to the low internet-users. The immediate negative impact of exposure to the internet on the mood of internet addicts may contribute to increased usage by those individuals attempting to reduce their low mood by re-engaging rapidly in internet use.

## Introduction

Over the past decade, since the term became widely debated in the medical literature [1], ‘internet addiction’ has become regarded as a novel psychopathology [2] that may well impact on a large number of individuals [3]. The focus of internet use in ‘internet addicts’ is varied, but using the internet for gambling [4] and pornography [5] are common amongst such individuals. The negative impact of excessive internet use can be seen across a wide range of aspects of the persons’ life [6], [7], as well as on many aspects of their family functioning [8]. However, there has been virtually no research exploring the immediate psychological impacts of internet exposure on ‘internet addicts’, which can act as a driver of such problematic behaviour.

It is known that individuals who could be classed as ‘internet addicted’ manifest a range of co-morbid psychological symptoms [9], such as depression [10], [11], attention deficit and hyperactivity disorder [5], [10], as well as social isolation and low self-esteem [12]–[14]. Moreover, they can also display a range of personality characteristics and traits [15], such as impulsivity [16], sensation- and novelty-seeking [17], [18] and sometimes enhanced levels of aggression [19], [20]. Although these findings regarding the characteristics of those who may be at risk of internet addiction are informative, establishing a model that involves the proximal (e.g., motives and reinforcement), as well as the distal causes of internet addiction is paramount in developing understanding and treatments of the disorder [21]–[23]. To this end, the current study explored whether exposure to the internet differentially immediately impacts the psychological states of internet addicts compared to those who do not display problematic internet behaviour.

It is often assumed that internet use is maintained by the positive reinforcing consequences of such use; for example, its production of entertainment, use as a pass-time, or in information-seeking [13]. Moreover, it has been suggested that high use may be motivated by factors such as identify-clarification, certainly in adolescent users [24]. However, it is often noted that other psychological factors, not linked to positive reinforcing consequences, are often implicated in maintaining high levels of problematic behaviours. For example, exposure to situations involving risk do not provoke increased anxiety in those who display problematic gambling behaviours [4], [25]. Similarly, exposure to the object of the problematic behaviours has been found to reduce mood [26], especially in individuals addicted to pornography [5], [27]. As both of these reasons (i.e. gambling and pornography) for use of the internet are strongly associated with problematic internet use [2], [3], [14], it may well be that these factors may also contribute to internet addiction [14]. Indeed, it has been suggested that such negative impacts of engagement in problematic behaviour may, in themselves, generate further engagement in these high probability problematic behaviours in an attempt to escape these negative feelings [28].

However, as very little is currently known about the immediate psychological impact of internet exposure on those with problematic internet behaviours, the development of models, let alone appropriate interventions, is still difficult. Given this, the current study explored whether exposure to the internet differentially impacted the psychological state of high- and low internet-users. To this end, the sample was assessed for the extent to which their internet use disrupts their everyday life. Participants’ mood and anxiety were then measured, they were then allowed access to any websites that they wished, and then were re-assessed for their levels of mood and current anxiety to determine if exposure to the internet had different effects on internet addicts to those without such problematic behaviours. In addition, to ensure compatibility with previous investigations of the characteristics of problematic internet users [11], [12], [17], [19], this study also explored the associations between internet addiction and other psychological symptoms. Participants were given a battery of psychological tests to assess their long-standing anxiety levels and depression. In addition, novel measures in this context of co-morbidity involving schizotypy and autism-like traits were assessed, as both psychosis [14] and social-isolation [12] have been associated with internet addiction previously.

## Methods

### Ethics Statement

Ethical approval for this research was obtained from the Department of Psychology Ethics Committee, Swansea University. The participants provided their written informed consent to participate in this study, and the Ethics Committee approved this consent procedure.

### Participants

Sixty volunteers responded to a request for participation in a psychology study, which was advertised on and around Swansea University campus. There were 27 males and 33 females, with a mean age of 24.0+2.5 years. None of the participants received any payment for their participation.

### Materials

***Internet Addiction Test*** (IAT) [29] is a 20-item scale covering the degree to which use of internet disrupts everyday life (work, sleep, relationships, etc.), the score ranges from 20 to 100. The internal reliability of the scale is 0.93.

***Positive And Negative Affect Schedule*** (PANAS) [30] is a 20-item questionnaire designed to measure participants’ positive and negative moods. Participants are required to choose the number that corresponds to the intensity of their feeling concerning the item, ranging from 1 = very slightly to 5 = extremely), and the total scores can range from 10–50. The internal reliability of both the positive and negative scales is 0.90.

***Spielberger Trait-State Anxiety Inventory*** (STAI-T/S) [31] rates the affective, cognitive, and physiological manifestations of anxiety in terms of long-standing patterns (trait anxiety) and current anxiety (state). The total score for each scale ranges from 20 to 80. The internal reliability of the scale is 0.93.

**Beck’s Depression Inventory** (BDI) [32] is a 21-item questionnaire that assesses the clinical symptoms of depression through asking about feelings over the past week. The score ranges from 0 to 63. The internal reliability of the scale is 0.93.

***Oxford Liverpool Inventory of Feelings and Experiences - Brief Version*** (O-LIFE(B)) [33] is a 43 item scale consisting of four subscales (unusual experiences, cognitive disorganization, introvertive anhedonia and impulsive non-conformity) designed to measure schizotypy in the normal population. The scales have an internal reliability between 0.72 and 0.89.

***Autistic Spectrum Quotient*** ** ***Questionnaire*** (AQ) [34] measures the level of autistic traits that an individual lacking an ASD diagnosis may possess. This questionnaire consists of 50 questions, with a score of 32 generally being suggested as indicating Asperger’s syndrome or high functioning autism. The internal consistency of the scale is 0.82.

### Procedure

The participants were seated alone in a quite room and tested individually. After a brief introduction to the study, they were asked to complete the battery of psychological tests (given in random order to the participants, with the exception that the PANAS and STAI-S which were always completed last). After completing the tests, the participants were allowed access to the internet through the computer in the room for 15 mins. The content of the sites that they visited was not recorded in this study, and the participants were told explicitly that this would be case. This procedure was adopted to encourage them to visit whatever site they may wish, irrespective of whether the content of that site might be regarded as socially appropriate. After 15 min they were asked to complete the PANAS and STAI questionnaires again.

## Results

Table 1 shows the means (standard deviations) for all psychometric measures taken prior to internet exposure, and their Spearman correlation coefficients with the internet addiction test (IAT). Inspection of the means shows that the sample as a whole fell within the expected range for these psychometric assessments. The Spearman’s correlations revealed strong associations between internet addiction and depression (BDI), schizotypal impulsive nonconformity (OLIFE IN), and also with autism-traits (AQ). There were also weaker associations between internet addiction and long-standing anxiety (STAI-T), and negative mood (PANAS-).

**Table 1:** Means (standard deviations) for all psychometric measures and their spearman correlation coefficients with internet addiction test (IAT).

Scale		Internet Addiction (IAT)
	Mean (SD)	40.6+12.9
Depression (BDI)	7.5+6.4	.415***
Positive Mood (PANAS+)	30.3+6.3	.226
Negative Mood (PANAS-)	13.8+4.2	.255*
State Anxiety (STAI-S)	34.7+11.2	.240
Trait Anxiety (STAI-T)	39.5+11.4	.298*
Schizotypal Unusual Experiences (O-LIFE)	4.0+3.2	.221
Schizotypal Cognitive Disorganisation (O-LIFE)	4.3+2.8	.262*
Schizotypal Introverted Anhedonia (O-LIFE)	2.8+1.8	.231
Schizotypal Impulsive Nonconformity (O-LIFE)	2.9+1.7	.394**
Autism Traits (AQ)	15.8+6.2	.404***

* *p*<.05;

** *p*<.01;

*** *p*<.001.

The sample was then divided at the mean for the IAT score to produce groups of lower- and higher-problematic internet use groups; the mean for the IAT was 41, which is also taken to reflect some degree of problematic usage [13]. This produced a lower-problematic use group (*n* = 28, mean = 29.5+7.9; 13 male, 15 female), and a higher-problematic use group (*n* = 32, mean 50.3+7.2; 18 male, 18 female).

**Figure 1:**
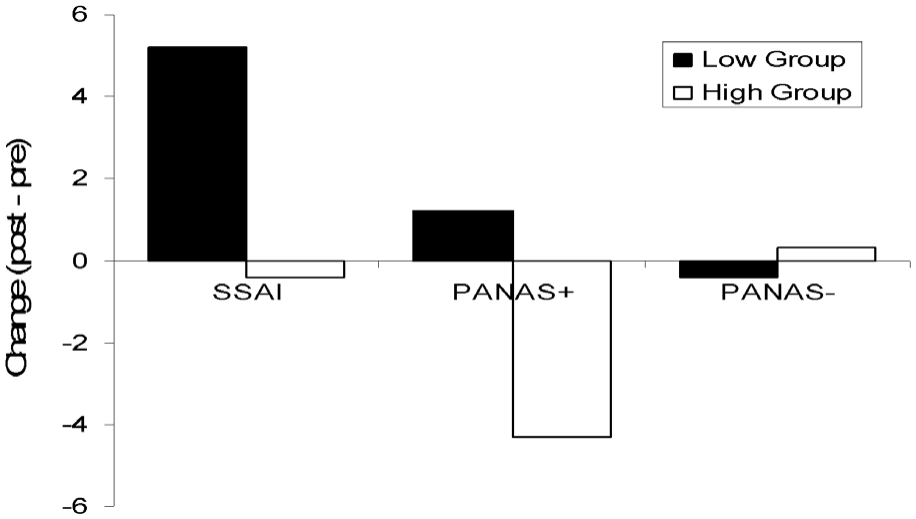
Shows the change between post- and pre-internet use in state anxiety (SSAI), positive mood (PANAS+), and negative mood (PANAS-) for both the low internet-using (Low) and high internet-using (High) groups.

Figure 1 shows the change, relative to pre-internet use, in state anxiety (SSAI), positive mood (PANAS+) and negative mood (PANAS-) immediately after exposure to the internet for the two groups. There was a significantly greater increase in current anxiety for the lower-problem group compared to the higher-problem group, Mann-Whitney *U* = 318.5, *p*<.05; the low-using group showing increased anxiety relative to pre-internet use, Wilcoxon *z* = 2.09, *p*<.05, but no change for the high-using group, *p*>.70. There was a significantly greater drop in positive mood for the higher-problem use group compared to the lower-problem group, *U* = 234.0, *p*<.001; the low-user group showing no change relative to baseline, *p*>.20, but the high-user group showing strongly decreased positive mood, *z* = 3.31, *p*<.001. There was no significant impact of internet exposure on negative mood for either group, all *p*s >.10.

## Discussion

The current study aimed to explore the potential differential impact of internet exposure on ‘internet addicts’ compared to those with little problematic usage. The results showed a striking negative impact of internet exposure on the positive mood of ‘internet addicts’. This effect has been suggested in theoretical models of ‘internet addiction [14], [21], and a similar finding has also been noted in terms of the negative effect of exposure to pornography on internet sex addicts [5], which may suggest commonalities between these addictions. It is also worth suggesting that this negative impact on mood could be considered as akin to a withdrawal effect, suggested as needed for the classification of addictions [1], [2], [27]. This finding suggests that, as with other forms of problematic behaviours [5], [21], excessive internet usage may be escape-maintained [14] and self-fuelling – engagement in the behaviour lowers mood, which then triggers further engagement to escape from the low mood [21]. The lack of impact on anxiety seen in problematic internet-users of exposure to the internet is also observed in problematic gamblers on exposure to risk-laden situation [4], [25], and again suggests commonalities between internet addiction and other forms of problematic behaviours.

It should be pointed out that, as two of the key uses of the internet for a sizable number of internet users are to gain access to pornography and gambling [4], [5], and these latter activities are clearly subject to potentially-addictive states, it may be that any results relating to ‘internet addiction’ are actually manifestations of other forms of addiction (i.e. to pornography or gambling).

Apart from the demonstration of differential psychological impacts of internet exposure on ‘internet addicts’, there were a number of findings that were worthy of comment. The associations between internet addiction and depression [10], [11], and schizotypal impulsive nonconformity [14], [17] are already known, and demonstrate that the current sample is similar to those previously studied. However, that internet addiction was strongly related to autistic traits is a novel finding, and may be similar in nature to previously established associations between social isolation and internet addiction [12]. This latter finding is potentially interesting and worthy of further study, but the reasons for this association are currently unclear. It may be that those with higher traits of autism engage in the internet more as a preferred method of interaction. In which case, higher internet use may not be problematic in this group. Alternatively, engagement in the use of internet may be an isolated activity by nature, and, the degree to which this occurs, and the participant is, in this manner, often in situations of social isolation, may impact on the responses given to the autism scale, giving a spurious association with autistic traits. Clearly further work is needed in this area.

Apart from these findings related to the psychological characteristics of those with problematic internet usage, two features of the current data are noteworthy. Firstly, over 50% of the sample (32/60) produced scores on the IAT that could be considered to represent some degree of problematic behaviour [26]. This may represent a function of recruiting the sample from younger people on a university campus, but, if replicated, would suggest a level of problem hereto not suggested. The gender split of those with problematic internet use versus those without was even, suggesting that typical views of internet addiction as a male problem are (certainly, now) unfounded.

There are a number of limitations of the present study that should be mentioned, and which could be addressed in subsequent research. In this experiment, participants were given only 15 min exposure to the internet, and the impact of this exposure on their mood was assessed. Although this length of exposure is enough to produce an impact on mood, as measured by the current scales, it is not known what longer exposure times would, nor is the temporal dynamic of changes in mood and anxiety during exposure to the internet currently known. Moreover, the content of websites visited by the participants during their period of exposure was not monitored in this investigation. This was done to encourage the participants to freely explore the internet in any way that they wished. However, as it is not certain what sites the participants did visit, it cannot be concluded that these would be the typical sites that they use the internet to explore. Of course, if these sites included those with pornographic or gambling content it is unlikely that these would be visited in the current context. Indeed, it is not clear that such sites would be reliably reported as being visited in the context of any such study. However, given this limitation, it is still not known whether the impacts on mood obtained in this context would similarly be observed in other contexts of use, and this remains an area in need of study.

Taken together with previous findings, these results help to build a picture of the distal and proximal causes of excessive internet usage. Certainly, those with long-standing depression [11] and anxiety [12], coupled with social isolation [13], and a lack of anxiety about novel technologies [17], [19], may be at risk from excessive internet usage [3], [21]. However, the subset of those individuals who then experience a negative impact on positive mood after internet exposure may then be triggered into further escape-motivated internet use, suggesting a possible mechanism maintaining internet use in internet addicts.
